# P-1764. Point Prevalence Study of Antibiotic Appropriateness and Economical Cost in a Tertiary Care Trauma Hospital of Northern India to Tailor Antibiotic Stewardship Interventions

**DOI:** 10.1093/ofid/ofae631.1927

**Published:** 2025-01-29

**Authors:** Rahul Garg, Archana B Wankhade, Tuhina Banerjee

**Affiliations:** AIIMS Bibinagar, Hyderabad , Telangana, India; AIIMS RAIPUR, Raipur, Chhattisgarh, India; Institute of Medical Sciences, BHU, Varanasi, VARANASI, Uttar Pradesh, India

## Abstract

**Background:**

Conducting Point prevalence surveys (PPSs) is challenging, especially in developing countries, in the absence of electronic records, lack of dedicated resources and high patient load. This survey was conducted to provide background data for strengthening antimicrobial stewardship(AMS) practices and estimate the economic cost of antimicrobial resistance per antibiotic consumed in a tertiary care trauma hospital in India.

Summary of Quality indicators of antibiotic prescribing (in %)

**Figure:**
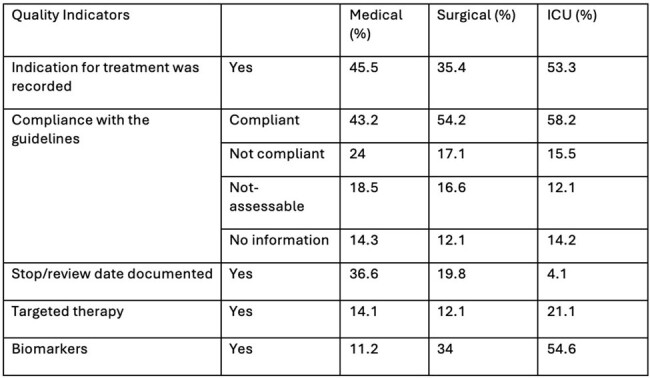

**Methods:**

The survey included all inpatients admitted in wards and intensive care units of the hospital (300 beds) during World Antibiotic Awareness Week 2023. Standardised modules were adopted from the Global PPS for collecting data on patient demographics, prescribed antimicrobial agents with dosage, indication(s) and duration of treatment, and microbiolgical results. Further, the cost minimization analysis was carried out to assess the treatment cost and variations in costs arising due to generic or branded prescribing.

**Figure:**
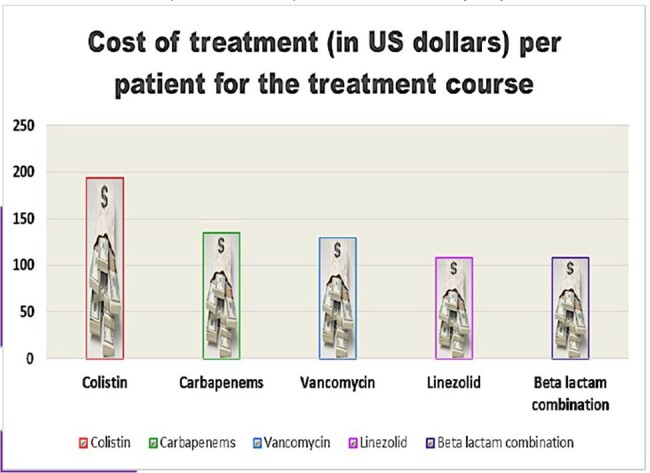

**Results:**

Among the 254 patients admitted, the patients treated with at least one antimicrobial was 88.6% (N=225). The median (IQR) day of hospitalization was 7 (3.75) days. The highest prevalence of antimicrobial use was in the ICU (100%), followed by Emergency medicine (97.5%). The majority of patients received empiric than targeted antimicrobial therapy. Antimicrobial prescription was mostly for surgical prophylaxis (49.04%). Among patients for >7 days on treatment, microbiological culture were sent in 16.7% (5/30) patients. Antibiotic quality indicators such as reason in notes and documentation of review date were comparatively lower (Figure-1). Treatment costs varied significantly with prescriptions of maximum and minimum-priced brands, ranging from 69.81%-1490%. Branded therapy were three times costlier than generic therapy in 36 (52.9%) regimens.

**Conclusion:**

Poor transition from empiricism to rationalism, continuation of surgical prophylaxis for more than one day and underutilization of microbiological tests were noted. There was large variations in treatment cost of infections depending on regimen chosen. Adherence to local antibiotic policy, optimal dosing and regular audit with positive feedback of antibiotic use to treating physician can be useful approaches to implement the AMS Program in the study centre.

**Disclosures:**

**All Authors**: No reported disclosures

